# Supplementary material for: Biopsychosocial risk factors and knowledge of cervical cancer among young women: A case study from Kenya to inform HPV prevention in Sub-Saharan Africa
Source: PLoS One. 2020 Aug 20;15(8):e0237745. doi: 10.1371/journal.pone.0237745 (PMC7446823; doi:10.1371/journal.pone.0237745)
Supplement: S2 File — (PDF) [file pone.0237745.s002.pdf]

From: archive@dhsprogram.com
To: imarigu@gmail.com
Subject: DHS Download Account Application
Date: Monday, 16 March 2020 4:25:14 AM
Attachments: AuthLetter_140376.pdf

Please see the attached authorization letter.

You are authorized to download Survey data from the Demographic and Health Surveys (DHS) Program. The DHS Data may be used only for the purpose of statistical reporting and analysis, and only for your registered research. To use the data for another purpose, a new research project must be registered. All DHS data should be treated as confidential, and no effort should be made to identify any household or individual respondent interviewed in the survey. Please reference the complete terms of use at: https://dhsprogram.com/Data/terms-of-use.cfm.

The data you download, must not be passed on to others, but you may share the data with coresearchers registered on this project.

Coresearcher(s):
Fatch Kalembo: fatchwelcom.kalembo@curtin.edu.au
Barbara Lossel: Barbara.Loessl@murdoch.edu.au
Lucy Bitok: Lukibitok@uonbi.ac.ke

All other interested users are required to register for a download account, and access the data from their approved accounts. Users are required to submit an electronic copy (pdf) of any reports/publications resulting from using the DHS data files to: references@dhsprogram.com.

To begin downloading datasets, please login at: The DHS Program login page or the IPUMS DHS login page.

THE DHS PROGRAM

The files you will download are in zipped format and must be unzipped before analysis. After unzipping, please print the file with the Word document (found in the Individual and Male Recode Zips). This file contains useful information on country specific variables and differences in the Standard Recode definition. You will also need the Guide to DHS Statistics, the questionnaires used in the survey, and the DHS Recode Manual. The Recode Manual contains a general description of the recode data file, including the rationale for recoding; a description of coding standards and recode variables, and a listing of the standard dictionary, with basic information relating to each variable.

The types of datasets generated for each survey vary by survey design. However there are seven common types of recode data files associated with the core questionnaires. The three questionnaires: the household, the woman's and the men's; from an analytical point of view, contain the analytical units of: household information, household member's information, women's information, children's information (of the interviewed women), and men's information. Further, the children's information exists in two groups – basic data for all children of a woman, and more in depth information for children born in the last five years. Then, where possible, individual men and woman are matched into couples. Listing of files:

- Household file (HR)
- Household members, or persons file (PR)

- Women’s file (IR)
- All Births file (BR)
- Children born in the 5 years prior to the interview, or kids file (KR)
- Men’s file (MR)
- Couple’s file (CR)

IPUMS DHS

If you use **IPUMS DHS**, you will have the option of choosing your record type (e.g., women, household members) when you begin selecting data. Variable names are links that provide extensive information, including variable descriptions, cautionary notes, and the survey text used to generate each variable. Variables in IPUMS are harmonized across samples, making this an excellent tool for comparative analysis. You will download a single integrated dataset with all the samples and variables that you select. IPUMS DHS is currently available for Standard DHS surveys from many, but not all, DHS participating countries. More samples are being added all the time.

DHS METHODOLOGICAL INFORMATION

For additional methodological information about DHS data and data quality, please see:

- DHS Methodological Report series
- External Database of Journal Articles based on DHS data

SOME USEFUL DHS LINKS

- Model questionnaires
- Model DHS Datasets
- STATcompiler
- Final Country Reports
- File Types and Names
- DHS Recode Manual
- Guide to DHS Statistics
- DHS User Forum
- DHS Video Tutorials
- Using DHS Data for Analysis
- Merging DHS Datasets

We now have repositories of code written in Stata and SPSS available on Github. Please reference these code repositories as a resource for code to match or calculate DHS indicators. The code repositories can be found at:

- https://github.com/DHSProgram/DHS-Indicators-Stata
- https://github.com/DHSProgram/DHS-Indicators-SPSS

For problems with your user account, please email archive@dhsprogram.com. For problems using the IPUMS DHS website, please email ipums@umn.edu. For data related questions, please register to participate in the DHS Program User Forum at: http://userforum.dhsprogram.com.

The Demographic and Health Surveys (DHS) Program
ICF
530 Gaither Road
Suite 500
Rockville, MD 20850

USA

LOGIN INFORMATION:
Login Email: imarigu@gmail.com
Password: (use password selected when you registered)

Name: Irene Ngune
Project: Cervical cancer awareness among the youth in Kenya: A case study
