# Supplementary material for: Biopsychosocial risk factors and knowledge of cervical cancer among young women: A case study from Kenya to inform HPV prevention in Sub-Saharan Africa
Source: PLoS One. 2020 Aug 20;15(8):e0237745. doi: 10.1371/journal.pone.0237745 (PMC7446823; doi:10.1371/journal.pone.0237745)
Supplement: Attachment — Submitted filename: Rejoinder - Final.docx [file pone.0237745.s003.docx]

**Rejoinder:**

Manuscript: Biopsychological risk factors and knowledge of cervical cancer among young women in a case study in Kenya to inform HPV prevention in Sub-Saharan Africa

PLOS Manuscript Number PONE-D-20-14074

| Comments | Rejoinder |
| --- | --- |
| Reviewer 2 |  |
| 1. Background |  |
| a. These results just support already existing empirical knowledge about barriers to cervical cancer prevention programs in Kenya as referenced (reference 13 and 14). It also is consistent with the recommended targets of community mobilisation as outlined in chapter 3 of the WHO comprehensive cervical cancer control: a guide for essential practice publication (WHO 2014). However, the WHO guide is not mentioned. | *We have addressed this in the manuscript by adding the following part in background information :*  Our study builds on the findings of previous studies on cervical cancer in Kenya and is also consistent with the recommended targets of effective cervical cancer prevention such as community mobilisation, outreach, and health education as outlined in chapter 3 of the WHO comprehensive cervical cancer control guide[1] (*See page 6, last paragraph).* |
| b. The weakness of the study is grounded in the not clear justification why this general national wide analysis will be important for cervical cancer prevention program design and management to improve the knowledge about cervical cancer as all these factors are already well known. | *The authors appreciate the reviewer’s comments and concur that further justification of the study was needed. We have addressed this by adding the following part in the background and discussion sections:*  Studies conducted in Kenya on determinants of cervical cancer awareness have provided results for women of the entire reproductive age-group (15-49 years), with limited attention on the pre-screening age-group (15-24 years). Similarly, primary HPV vaccination in Kenya has targeted adolescents aged 9-14 years [2, 3]; hence the 15-24-year-olds are less catered for by programs.  Young women aged 15-24 years, would be an ideal target group for behavioural interventions to prevent cervical cancer in Kenya because of their developmental stage and also the fact that most are sexually active [4]. (*Please see background section, page 6 paragraph 2)*  ‘An interesting finding, which is different from the findings of the previous studies related awareness of cervical cancer in Kenya was the fact that the majority of young women (87%) felt that they were justified to ask their partners with an STI to use a condom, but only 25% thought they actually could demand it. The low self-efficacy in women with regards to safe sex practices predicted higher levels of unawareness of cervical cancer. Our study has shown that behavioural issues related to self–efficacy such as inability to refuse unsafe sex, is a significant determinant of cervical cancer awareness for this age-group. Self-efficacy to cervical cancer prevention among Kenyan women has not been extensively covered due to a lack of population-based studies. A qualitative study by Ngugi et al. 2012 on factors affecting uptake of cervical cancer screening in Kenya, reported self-efficacy in relation to attendance of screening among older women [5]. The results of this study did not explicitly inform self-efficacy of the pre-screening age (*Please see the discussion section, page 23 paragraph 1)* |
| c. Epidemiology of cervical cancer in Kenya (page 4-6). The authors are quoting the IARC world cancer report without being more specific about the chapter/page they are referring to. | *The manuscript has been amended accordingly to include the page number*  A report by International Agency for Research on Cancer, 2015, page E373, showed that of the cancers that affect women in East Africa, that cervical cancer has the highest number of new cancer cases (45.7/100,000), followed by breast cancer (33.5/100,000) and oesophageal cancer (9.8/100,000) [6] (*Please see the introduction section, page 4, second last paragraph).*  Ferlay J, Soerjomataram I, Dikshit R, Eser S, Mathers C, Rebelo M, et al. Cancer incidence and mortality worldwide: Sources, methods and major patterns in GLOBOCAN 2012. International Journal of Cancer. 2015;136(5):E359-E86. doi: 10.1002/ijc.29210 *(please see reference 5 page 27).* |
| d. The term “prevalence” should not be linked to data which show incidence figures. | *The text has been rephrased to read as below:*  A report by International Agency for Research on Cancer, 2015, page E373, showed that of the cancers that affect women in East Africa, that cervical cancer has the highest number of new cancer cases (45.7/100,000), followed by breast cancer (33.5/100,000) and oesophageal cancer (9.8/100,000) [6] (*Please see the introduction section, page 4, second last paragraph)* |
| e. The term high risk HPV types need to be introduced. | *The manuscript has been amended to introduce HR-HPV types.*  Cervical cancer is mostly caused by a persistent infection with twelve specific carcinogenic types of human papillomavirus (HPV) which is transmitted sexually by males and females shortly after the onset of sexual activity [7, 8]. However, the two most common high-risk HPV (HR-HPV) that cause cervical cancer in women are 16 and 18. The HR-HPV 16 and 18 are responsible for approximately 70% of cervical cancers worldwide [9] (*Please see the introduction section, page 4, last paragraph)* |
| f. Again, the IARC world cancer is referred as source for information about sexual risk behaviour without being specific about chapter and page. | *The references have been amended to include the chapter and page numbers*  “14. Wild C, Weilderpass, E., Stewart, B. World Cancer Report: Cancer Research for Cancer: Etiology: Human Papilloma Virus Chapter 5.2, page 317. International Agency for Research on Cancer, 2020”  ‘15. Wild C, Weilderpass, E., Stewart, B. World Cancer Report: Global trends in cancer incidence and mortality, Chapter 1.2, page 31. International Agency for Research on Cancer, 2020.’ (*Please see references 14 and 15).* |
| g. Late presentation of cervical cancer is supported by a reference 5 which however, is analysing breast cancer late presentation. | *We have addressed this in the manuscript by adding the following part in background information*  Multifactorial biopsychosocial risks lead women to delay screening and treatment [10, 11]. A study on late presentation of women with cervical cancer conducted in Kenya showed that the main reasons for poor access were poverty, lack confidence in orthodox medicine, and lack of access to health care [10] (*Please see page 5, last paragraph).* |
| 2. Materials and Methods |  |
| a. It is unclear why the authors have chosen the age group 15- 24, means a heterogeneous group of adolescent girls and young women. In accordance with the national guidelines (Box 1), the adolescent girls are beyond the target group of HVP vaccination programs and the young women are too young for screening programs. | *To optimise clarity as to why this age-group was chosen, the manuscript has been revised accordingly:*  The findings of this study will inform the development of cervical cancer prevention strategies that are specific to this group as evidence shows that women are less likely to develop cervical cancer when preventive interventions are implemented before the age of 26 [12] (*Please see the introduction section last paragraph, page 6).*  This particular age-group was selected because they are in the cervical cancer pre-screening age and understanding factors that affect their awareness of cervical cancer would be necessary for planning prevention programs for this age group. Also, majority of programs and research in Kenya target adolescents (9-14 years) [13] and women of entire reproductive age-group [5, 11, 14] indicated that this group is less catered for (*Please see the methods section last paragraph, page 10).*  Moreover, with the introduction of the ‘100% transition policy’ by the Kenyan government for primary school students to attend high school, prevention programs targeting 15 to 24 year olds could easily be rolled out through high schools and colleges [15] (*Please see the discussion section paragraph 1, page 24).* |
| b. It would be interesting to know the level of knowledge among women in the screening target group (25 – 49) and the level of knowledge of the mothers of the adolescent girls. | *This has been acknowledged in the study and has been added as an area for future research (see the paragraph below, which is also on page 21, first paragraph:*  A significant proportion of young women in our study had never heard of cervical of cancer (32.4%). The proportion of women who had low levels of cervical cancer in this study is higher than those reported by authors of two previous Kenyan studies (20%) [13] and 24% [14]. The difference in levels of knowledge between our study and the other two Kenyan studies is likely to be due to differences in the age of the participants. While our study targeted the 15 to 24 years old, young women, the other two Kenyan studies targeted women of reproductive age group (15-49 years) [13-14]. Future studies in Kenya should assess the levels of knowledge of cervical cancer among women aged between 25 to 49 years, as this is the recommended age group for cervical cancer screening programs in Kenya. |
| c. Education level as a summary measure in the group 15 – 24 might be misleading because the group is in remarkably diverse individual development stages. This also applies to sexual activity and self-efficacy in prevention behaviour. | The study compared both age-groups (15-19 & 20-24 age groups) in the preliminary analysis, and there were no significant differences in the knowledge levels on the selected variables, including education level. The authors refer to the following text:  ‘Close to one-third of the participants were unaware of cervical cancer with no difference between participants aged 15-19 years (n=2716) and those aged 20-24 years (n=2691) (OR=1; CI = 0.69 – 1.45).  *Please see abstract page 2 and Table 2, page 15.* |
| 3. Discussion |  |
|  |  |
| a. More discussion and reflection would be expected about the relationship between low level of knowledge among the study group (15-25) and how this relates to the known low- level of screening uptake in older age groups which assessed in Kenya according to reference 14. | *This has been acknowledged in the study and has been added in the discussion section – page 21*  A significant proportion of young women in our study had never heard of cervical of cancer (32.4%). The proportion of women who had low levels of cervical cancer in this study is higher than those reported by authors of two previous Kenyan studies (20%) [13 and 24% [14]. The difference in levels of knowledge between our study and the other two Kenyan studies is likely to be due to differences in the age of the participants. While our study targeted the 15 to 24 years old, young women, the other two Kenyan studies targeted women of reproductive age group (15-49 years) [13-14]. Future studies in Kenya should assess the levels of knowledge of cervical cancer among women aged between 25 to 49 years, as this is the recommended age group for cervical cancer screening programs in Kenya. |
| b. The low up adherence to screening among eligible women in Kenya might depend on many other factors thank knowledge such as availability of screening services. | The manuscript has been revised accordingly and now reads as follows:  This would necessitate education campaigns to make all women aware, a roll-out strategy for those secondary immunisations, and the financial ability to do so. Additionally, screening needs to be available for those who have not been vaccinated to allow early diagnosis and treatment. HPV testing is the gold standard for screening but is not always possible to conduct due to financial and logistical reasons [7, 16]. Other challenges that lead to low uptake screening services include fear of being diagnosed with the disease, long distance to the health facility, and shame of undergoing a vaginal examination [1]. Thus, service providers, policymakers and researchers need to consider these challenges when planning cancer preventive interventions (*See page 23 last paragraph).* |
| c. It is unclear why there is mentioning that a proxy variable “Ever completing a HIV test” was used to assess awareness of cervical cancer! It might be a typo. | The authors acknowlsge the error and have amended the manuscript to read as follows:  We assessed the effect of biological factors of age, parity and age at first sexual contact but did not investigate other clinically relevant factors such as HIV status that are associated with increased prevalence of cervical cancer [17]. Our study did, however, use the variable of ‘ever completing an HIV test’ to assess awareness of cervical cancer that has also been used in similar studies that have used the KDHS data.  *Please see page 26 paragraph 1* |
| 4. Summary of the issues |  |
| a. The results of the evaluation of the subgroup of 15 – 24 year old adolescent and girls within the KDHS survey are not new and do not bring any new dimensions which need to be addressed in HPV vaccination and cervical cancer prevention programs. It is therefore not clear how this helps in further refining the national cervical cancer program in Kenya. It might be useful to do analysis at the level of the 47 counties where the survey was performed to identify local difference in knowledge and communities which need special attention. | The authors have addressed the new dimensions this study brings to cancer prevention in sections 1b and 2a of the rejoinder.  Also, the county variable was not used during the analysis due to the constant changes of counties’ geographical boundaries since the 2008 KDHS data. Instead, a more stable variable (rural/urban) was used. |

**References**

1.	WHO. Comprehensive cervical cancer control: A guide to essential practice – 2nd ed, Chapter 3 page 81-100. Geneva, Switzerland: 2014 Contract No.: ISBN 978 92 4 154895 3.

2.	Mugo NR, Eckert L, Magaret AS, Cheng A, Mwaniki L, Ngure K, et al. Quadrivalent HPV vaccine in HIV-1-infected early adolescent girls and boys in Kenya: Month 7 and 12 post vaccine immunogenicity and correlation with immune status. Vaccine. 2018;36(46):7025-32. doi: 10.1016/j.vaccine.2018.09.059.

3.	Mabeya H, Menon S, Weyers S, Naanyu V, Mwaliko E, Kirop E, et al. Uptake of three doses of HPV vaccine by primary school girls in Eldoret, Kenya

a prospective cohort study in a malaria endemic setting. BMC Cancer. 2018;18(1). doi: 10.1186/s12885-018-4382-x.

4.	Okigbo CC, Speizer IS. Determinants of Sexual Activity and Pregnancy among Unmarried Young Women in Urban Kenya: A Cross-Sectional Study. PloS one. 2015;10(6):e0129286-e. doi: 10.1371/journal.pone.0129286. PubMed PMID: 26047505.

5.	Ngugi CW, Boga H, Muigai AW, Wanzala P, Mbithi JN. Factors affecting uptake of cervical cancer early detection measures among women in Thika, Kenya. Health care for women international. 2012;33(7):595-613.

6.	Ferlay J, Soerjomataram I, Dikshit R, Eser S, Mathers C, Rebelo M, et al. Cancer incidence and mortality worldwide: Sources, methods and major patterns in GLOBOCAN 2012. International Journal of Cancer. 2015;136(5):E359-E86. doi: 10.1002/ijc.29210.

7.	Wild C, Weilderpass, E., Stewart, B. World Cancer Report: Cancer Research for Cancer. International Agency for Research on Cancer, 2020.

8.	Chan CK, Aimagambetova G, Ukybassova T, Kongrtay K, Azizan A. Human Papillomavirus Infection and Cervical Cancer: Epidemiology, Screening, and Vaccination—Review of Current Perspectives. Journal of oncology. 2019;2019.

9.	WHO. Human Papillomavirus 2020 [cited 2020 10-07-2020]. Available from: <https://www.who.int/biologicals/areas/human_papillomavirus/en/>.

10.	Gatumo M, Gacheri S, Sayed A-R, Scheibe A. Women’s knowledge and attitudes related to cervical cancer and cervical cancer screening in Isiolo and Tharaka Nithi counties, Kenya: a cross-sectional study. BMC cancer. 2018;18(1):745.

11.	Kangmennaang J, Onyango EO, Luginaah I, Elliott SJ. The next Sub Saharan African epidemic? A case study of the determinants of cervical cancer knowledge and screening in Kenya. Soc Sci Med. 2018;197:203-12. Epub 2017/12/19. doi: 10.1016/j.socscimed.2017.12.013. PubMed PMID: 29253722.

12.	Castle PE, Fetterman B, Akhtar I, Husain M, Gold MA, Guido R, et al. Age-appropriate use of human papillomavirus vaccines in the U.S. Gynecologic Oncology. 2009;114(2):365-9. doi: 10.1016/j.ygyno.2009.04.035.

13.	Moshi FV, Vandervort EB, Kibusi SM. Cervical Cancer Awareness among Women in Tanzania: an analysis of data from the 2011-12 Tanzania HIV and Malaria Indicators Survey. International journal of chronic diseases. 2018;2018.

14.	Mburu A, Itsura P, Mabeya H, Kaaria A, Brown DR. Knowledge of Cervical Cancer and Acceptability of Prevention Strategies Among Human Papillomavirus-Vaccinated and Human Papillomavirus-Unvaccinated Adolescent Women in Eldoret, Kenya. BioResearch Open Access. 2019;8(1):139-45. doi: 10.1089/biores.2019.0007.

15.	Anorlu RI. Cervical cancer: the sub-Saharan African perspective. Reprod Health Matters. 2008;16(32):41-9. Epub 2008/11/26. doi: 10.1016/S0968-8080(08)32415-X. PubMed PMID: 19027621.

16.	Kivuti-Bitok LW, McDonnell G, Abdul R, Pokhariyal GP. System dynamics model of cervical cancer vaccination and screening interventions in Kenya. Cost Effectiveness and Resource Allocation. 2014;12(1):26.

17.	Liu G, Sharma M, Tan N, Barnabas RV. HIV-positive women have higher risk of human papilloma virus infection, precancerous lesions, and cervical cancer. AIDS (London, England). 2018;32(6):795-808.
