# Supplementary material for: Biopsychosocial risk factors and knowledge of cervical cancer among young women: A case study from Kenya to inform HPV prevention in Sub-Saharan Africa
Source: PLoS One. 2020 Aug 20;15(8):e0237745. doi: 10.1371/journal.pone.0237745 (PMC7446823; doi:10.1371/journal.pone.0237745)
Supplement: Attachment — Submitted filename: Response to Reviewer.docx [file pone.0237745.s004.docx]

**Rejoinder**

Manuscript: Biopsychological risk factors and knowledge of cervical cancer among young women in a case study in Kenya to inform HPV prevention in Sub-Saharan Africa

PLOS Manuscript Number PONE-D-20-14074

|  |  |
| --- | --- |
| Reviewer Comments | **Response** |
| 1. Stating that only adjusted OR are shown is not entirely correct as your tables contain unadjusted OR. | The statement ‘Only multivariate results are described below.’ Has been removed - please see page 15 paragraph 1 |
| 2. Tables: there is no need for p-values, CI are sufficient and correct as you are presenting estimates. Please remove the stars and stick to CI only. | The p-values have been removed and the astrix denoting the levels of significance have also been removed. |
| 3. For Table 2 and 3 please indicate in the legend what the adjustment variables are. The tables need to be able to stand alone, with complete information. | The legends for Tables 2 and 3 have been revised as follows:  Table 2  Factors and covariates used in the computation are fixed for all variables in the table: Age in 5-year groups=20-24; If had a birth=No birth; Age at first sex=At first union; Type of place of residence=Urban; Highest educational level=Higher; Religion=No religion/other; Wealth index=Richer-Richest; Covered by health insurance=Yes; Current marital status=Widowed/divorced/separated; Getting medical help for self: getting money needed for treatment=No problem; Getting medical help for self: distance to health facility=No problem; Can get a condom=Yes; Ever been tested for HIV=Yes.  Table 3  Factors and covariates used in the computation are fixed for all the variables in the table: Getting medical help for self: getting permission to go=No problem; Frequency of reading newspaper or magazine=At least once a week; Frequency of listening to radio=At least once a week; Frequency of watching television=At least once a week; A healthy looking person can have HIV=Yes; Getting medical help for self: not wanting to go alone= No problem; Wife justified asking husband to use condom if he has STI=Yes; Can get HIV by witchcraft or supernatural means = No; Respondent can ask partner to use a condom=Yes. |
| 4. Discussion - please reconsider outlining future studies at the beginning of the discussion, this should be reserved for the final section, and consider all further research needs in combination. | The following text has been moved from the beginning of the discussion to page 24 last paragraph.  ‘While our study targeted the 15 to 24 years old young women, the other two Kenyan studies targeted women of the whole reproductive age group (15-49 years) [13-14]. Future studies in Kenya should assess the levels of knowledge of cervical cancer among women aged between 25 to 49 years as this is the recommended age group for cervical cancer screening programs’. |
| 5. Redo the spell check and check all brackets, e.g. L10 in the discsussion | A spell-check has been completed. Please see manuscript with track changes |
